# Biomimetic periosteum combining BMP-2-loaded M2 macrophage-derived exosomes for enhanced bone defect repair

**DOI:** 10.3389/fbioe.2025.1639394

**Published:** 2025-08-01

**Authors:** Feng Ling, Jianzhong Bai, Jile Xie, Jie Liu, Qifeng Lu, Lili Yuan, Hongye Li, Zhonglai Qian

**Affiliations:** ^1^ Department of Orthopaedics, The First Affiliated Hospital of Soochow University, Soochow University, Suzhou, China; ^2^ Department of Orthopaedics, Taizhou People’s Hospital, Taizhou, China; ^3^ Department of Orthopedics, The Second Affiliated Hospital of Shandong First Medical University, Tai’an, China; ^4^ Department of Orthopedic Surgery, Xuzhou Medical University and The Second People’s Hospital of Lianyungang, Lianyungang, China

**Keywords:** bone defect, exosomes, biomimetic periosteum, macrophage, BMP

## Abstract

Bone defect repair continues to present a significant clinical challenge due to the limitations of traditional grafting techniques and the complexity involved in establishing a conducive regenerative microenvironment. In this study, we described the development of a multifunctional biomimetic periosteum based on electrospun gelatin methacryloyl (GelMA) membranes functionalized with bone morphogenetic protein-2 (BMP-2)-loaded M2 macrophage-derived exosomes. This engineered periosteum replicated the structural orientation and functional properties of natural periosteum, thereby providing a synergistic approach to promoting bone regeneration. Our findings indicated that the biomimetic periosteum served as a biocompatible scaffold that supported cell adhesion, proliferation, and differentiation. The incorporation of M2 macrophage-derived exosomes facilitated the creation of an anti-inflammatory immune microenvironment by polarizing macrophages towards the M2 phenotype, while the sustained release of BMP-2 enhances osteogenic differentiation and mineralization. *In vivo* experiments using a rat cranial defect model demonstrated that the BMP-2@Exo-GelMA membrane significantly accelerated bone defect repair, achieving superior outcomes in new bone formation and vascularization compared to control groups. This study underscored the potential of integrating immunomodulatory and osteoinductive strategies to develop next-generation biomaterials for bone tissue engineering. The biomimetic periosteum represented a promising therapeutic approach for addressing critical-sized bone defects and advancing clinical practices in bone regeneration.

## 1 Introduction

Bone defects caused by trauma, degenerative diseases, congenital malformations or tumor resection have always been a clinical treatment challenge, especially in large-scale bone defects (Xue et al., 2025). The traditional techniques of autologous bone grafting and allograft bone grafting are often faced with the problems of donor shortages, immune rejection, and poor restoration results (El-Rashidy et al., 2017; Laird et al., 2021). As an important part of bone tissue, periosteum plays a key role in the repair process after bone injury (Raftery et al., 2018; Perrin and Colnot, 2022). Its outer layer consists of dense connective tissue, which provides mechanical protection and vascular support, while the inner layer is rich in bone progenitor cells, which can differentiate into osteoblasts and participate in bone regeneration under the appropriate environment (Gupta et al., 2021). The integrity and functionality of periosteum play a crucial role in vascularization, osteogenesis, and bone remodeling during bone regeneration, especially for large bone segmental bone defects (Zhang et al., 2022; Liu et al., 2020). In critical-sized bone defects, the periosteum is completely destroyed and difficult to recover, which is also a key issue in bone defect repair. In recent years, the development of bionic materials has provided new opportunities for bone repair, among which the electrostatic spinning technology has attracted much attention because of its ability to construct a nanofiber structure similar to periosteum (Zhao et al., 2024). This bionic periosteum can not only mimic the directional alignment properties of the periosteum, but also further promote the adhesion, proliferation and differentiation of osteoblasts by modulating its physical and chemical properties, which provides a new way to rebuild the function of the periosteum and promote the repair of bone defects (Lou et al., 2021; Wang et al., 2023a; Sun et al., 2023a).

Bone repair not only depends on the physical support of the periosteum, but also requires the creation of a favorable microenvironment for bone regeneration, in which immunomodulation is particularly important (Wang et al., 2024). Macrophages play an important role in this process, and their transformation from pro-inflammatory M1 to anti-inflammatory M2 is essential for bone regeneration (Lu et al., 2024; Xu et al., 2023). M2 macrophages not only secrete anti-inflammatory factors to alleviate local inflammation, but also promote the activity of osteoblasts by secreting osteogenic factors (Liu H. et al., 2023; Wang et al., 2022). Therefore, the strategy of promoting bone repair by regulating the direction of macrophage polarization has received much attention (Jin et al., 2019; Liu et al., 2024). For example, Xu et al. constructed a hybridized biphasic bionic periosteum to promote bone regeneration by regulating macrophages (Xu et al., 2023). In the early stage, this biomimetic periosteum can maintain a moderate inflammatory response of M1-type macrophages to the recruitment of mesenchymal stem cells and angiogenesis at the site of acute fractures. In the later stage, as the gel phase degraded, the IL-4 released by the biomimetic periosteum, in synergy with collagen, promotes the polarization of macrophages to the M2 type, which reconstructs the local microenvironment by secreting PDGF-BB and BMP-2, thereby promoting both vascular maturation and osteogenesis. Exosomes are nanoscale vesicles secreted by cells, with a particle size ranging from 30 to 150 nm (Welsh et al., 2024). Encased in a lipid bilayer, exosomes are enriched with specific membrane proteins (such as CD63 and CD9) and luminal components (including various proteins, nucleic acids, and lipids). As crucial intercellular messengers, exosomes transmit bioactive cargo and are extensively involved in diverse physiological and pathological processes, such as immune regulation and tissue repair (Yao et al., 2025). They also hold significant potential in disease diagnosis and therapy. Notably, exosomes derived from M2-type macrophages, as nano-tools for intercellular signal transmission, also have multiple advantages (Daneshvar et al., 2024).Their low immunogenicity, excellent biocompatibility, and potential as drug delivery carriers make them uniquely valuable in the field of bone regeneration (Jin J. et al., 2024; Jin S. et al., 2024).

Although creating a favorable microenvironment for bone regeneration is crucial, further promoting bone formation on this basis remains the key to accelerating bone defect repair. In recent years, many studies have integrated metal ions (such as magnesium, zinc, and copper), applied growth factors (such as VEGF), and stem cells into artificial periosteum materials to enhance the osteogenic ability of the periosteum (Wu et al., 2020). For instance, Mao et al. designed a novel biomimetic periosteum - bioactive glass fiber membrane loaded with zinc and magnesium ions, which can directly enhance osteogenic differentiation and vascularization through the slow release of metal ions, thereby accelerating bone regeneration (Mao et al., 2024). Additionally, Wu et al. constructed a VEGF-sustained-release hierarchical micro/nano-fiber biomimetic periosteum (Wu et al., 2020). This biomimetic periosteum mimics the microenvironment of the extracellular matrix, providing structural support for cell adhesion, proliferation, and differentiation, and promoting angiogenesis through the release of VEGF, ultimately facilitating the repair of bone injuries. Although these studies have improved bone repair to some extent, the effects of these approaches are often limited by insufficient local factors or low delivery efficiency, as well as serious side effects (such as inflammation and immune responses, excessive bone formation and ectopic ossification), which hinders their further application. Among the numerous factors promoting bone regeneration, bone morphogenetic protein-2 (BMP-2) has become the focus of research due to its potent osteogenic induction ability (Lou et al., 2025). BMP-2 can activate the differentiation signaling pathways of osteoblasts and significantly increase the formation rate of new bone tissue. However, the local efficient delivery of BMP-2 has always been a challenge (Raftery et al., 2018; Liu Y. et al., 2023). BMP-2 exhibits a short physiological half-life of approximately 7 min, which necessitates loading high doses into scaffolds to mitigate its inherent instability and rapid degradation. This approach, however, markedly elevates treatment costs and increases the likelihood of side effects such as inflammation, nerve injury, ectopic ossification, and potential tumorigenesis (Wang et al., 2023b).EV/EXO-based systems effectively overcome the limitations of conventional delivery strategies by offering natural tropism toward osteogenic cells, sustained and physiologically relevant release kinetics, and superior biocompatibility with minimal immunogenicity or cytotoxicity (Wang and Zhang, 2024). Therefore, combining the drug-carrying capacity of M2 macrophage exosomes and surface functionalizing them with biomimetic materials provides an innovative solution for the precise delivery.

**FIGURE 1:**
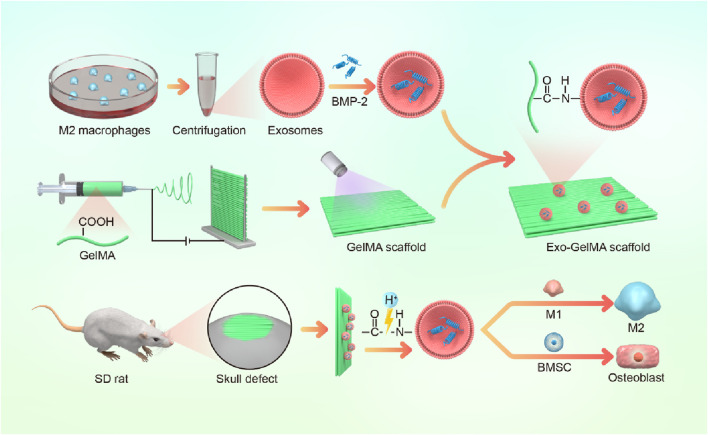
Schematic diagram of biomimetic periosteum promoting bone regeneration.

In summary, this study aimed to construct a multifunctional biomimetic periosteum based on GelMA electrospun membranes. BMP-2-loaded M2 macrophage exosomes were stably combined on the fiber surface through amidation reaction. This scaffold not only taked advantage of the immunomodulatory benefits of M2 macrophage exosomes but also achieved a synergistic effect of microenvironment optimization and osteogenesis enhancement through the local release of BMP-2 (Figure 1). We believe that this multifunctional biomimetic periosteum provides a novel engineered solution for bone defect repair and shows broad prospects for clinical translation.

## 2 Materials and methods

### 2.1 Preparation and characterization of biomimetic periosteum

Mononuclear macrophages extracted from the bone marrow of C57 mice (6 weeks old) were cultured in a culture dish. After 12 h, IL-4 (final concentration 20 ng/mL) was added, and the culture was continued for another 24 h to obtain M2-type macrophages. The exosomes of M2-type macrophages were extracted using an exosome extraction kit. The morphology and size distribution of the nanoparticles were characterized through transmission electron microscopy (TEM). Nanoparticle tracking analysis was employed to determine particle size. Western blot analysis was performed to detect the expression of specific exosomal markers including CD63 and CD81, as well as the cell lysate marker Calnexin. To determine the drug loading property of exosomes, recombinant human BMP-2 protein (10 mg/mL) was incubated with M2 macrophage-derived exosomes (1 × 10^6^ particles/mL) in a constant-temperature shaker at 37°C for 12 h. Then BMP-2-loaded M2 macrophage exosomes were obtained via centrifugation and washed 3 times with phosphate buffer solution (PBS). The BMP-2 loading efficiency was evaluated via BCA kit.

Dissolve 0.5 g of GelMA in 5 mL of hexafluoroisopropanol and mix thoroughly by heating and stirring at 40°C. Then, place the GelMA solution in a 5 mL syringe and fix it on a microinjection pump. Metal rods were placed parallel to the opposite side of the needle tip at a spacing of 4 cm, the voltage was set at 12 kV, and the flow rate was set at 2 mL/h to form parallel spinning fibers between the metal rods, obtaining the GelMA oriented electrospun scaffold.

Next, the electrospun scaffold (2 cm × 2 cm) was immersed in a solution of lithium phenyl (2,4,6-trimethylbenzoyl) phosphinate (LAP) photoinitiator, and after ultraviolet crosslinking, obtain the crosslinked GelMA electrospun scaffold. Dissolve 80 mg of EDC and 120 mg of NHS in 10 mL of MES solution to prepare the EDC/NHS activation solution. Immerse the crosslinked GelMA electrospun scaffold in the activation solution and incubate at 37°C for 15 min. Then, M2 macrophage exosomes (1 × 10^6^ particles/mL) were added and incubate at 37°C overnight. After washing with PBS, the Exo-GelMA biomimetic periosteum was obtained. The Fourier Transform Infrared (FTIR) and X-ray Photoelectron Spectroscopy (XPS) were performed to investigate the chemical composition and structural characteristics after surface modification. The microstructure and pore morphology of the bionic periosteum were observed using SEM. The membrane was subjected to tensile testing using a mechanical testing machine, and the shape variables of the membrane were recorded in real time during the test. Subsequently, the strain-stress curve was generated. To evaluate the ability of the biomimetic periosteum to release Exos, the membrane was immersed in PBS without Exos and other proteins. At predetermined time intervals, PBS samples were collected to assess the protein concentration.

### 2.2 Biocompatibility of biomimetic periosteum

BMSCs were extracted from the femurs of 6-8-week-old male SD rats. The experiment strictly followed the approval and guidance of the Animal Experiment Ethics Committee. The BMSCs were seeded onto the surface of the biomimetic periosteum and cultured for 24 h. The cells were stained with a Calcein/PI Cell Viability Assay Kit (Beyotime, Haimen, China) according to the protocol, and then observed under an inverted fluorescence microscope (Zeiss, Oberkochen, Germany). Green fluorescence indicated viable cells, while red fluorescence indicated dead cells.

The BMSCs were seeded onto the surface of the biomimetic periosteum and cultured for 24 h. The samples were fixed in 4% paraformaldehyde solution for 30 min and then permeabilized with 0.1% Triton X-100 solution for 10 min. Subsequently, they were stained with fluorescently labeled Phalloidin and DAPI for 30 min and 10 min, respectively. Finally, images were captured using an inverted fluorescence microscope (Zeiss, Oberkochen, Germany).

The BMSCs were seeded onto the surface of the biomimetic periosteum, and the proliferation of the cells was measured on days 1, 3, and 5. At each time point, 100 μL CCK-8 working solution was added to each well, and the cells were further cultured for 4 h. The absorbance (OD) values were measured at a wavelength of 450 nm using a PowerWave XS spectrophotometer (BioTek, Winooski, VT, United States).

The BMSCs were seeded onto the surface of the biomimetic periosteum and cultured for 24 h. After fixation with 4% paraformaldehyde solution and dehydration with gradient ethanol (10%, 30%, 50%, 70%, 85%, 90%, and 100%), the samples were observed under a SEM (S-4800, Hitachi, Kotyo, Japan).

### 2.3 Influence of biomimetic periosteum on macrophage polarization

Bone marrow macrophages (BMMs) were extracted from the femur of 6-week-old c57BL/6 mice. BMMs were co-cultured with the bionic periosteum for 24 h and then immunofluorescent staining was performed using specific antibodies labeled with M1 type (CD86, 1:500, ABclonal) and M2 type (CD206, 1:500, ABclonal) macrophage markers. Immunofluorescence staining was observed using fluorescence microscopy (Zeiss, Oberkochen, Germany).

Real-time fluorescence quantitative PCR (RT-PCR) was used to evaluate the effect of bionic periosteum on the expression of inflammatory genes (*Il-1β*, *Tnf*, *Il10* and *Tgfβ1*) in macrophages. After total cellular RNA was extracted, the RNA was reverse transcribed into cDNA using a reverse transcription kit. The reaction system included specific primers and SYBR Green dye. The reaction conditions were set according to the kit instructions, and the data were corrected using internal reference genes (GAPDH), and the relative expression was finally calculated by the comparative Ct (2^−ΔΔCT^) method. The primer sequences for the target genes are presented in Supplementary Table S1.

### 2.4 Effect of biomimetic periosteum on osteogenic differentiation ability

BMSCs were co-cultured with bionic periosteum for 7 days and then stained using alkaline phosphatase (ALP) staining kit (Beyotime, Haimen, China). ALP activity was observed by light microscopy, and its difference in each group was quantitatively analyzed.

The mineralization capacity of BMSCs was assessed using alizarin red staining (ARS). After co-culturing BMSCs with bionic periosteum for 14 days, ARS was performed using alizarin red staining working solution (Beyotime, Haimen, China), followed by photographic documentation with a light microscope. Calcium deposit was dissolved using potassium permanganate solution, and the level of mineralization was quantitatively assessed by measuring the absorbance value at 420 nm using a PowerWave XS spectrophotometer (BioTek, Winooski, VT, United States).

The effect of bionic periosteum on osteogenesis-related genes (*Runx2*, *Spp1*, *Col1a1*, and *Bglap*) was assessed using RT-PCR. The primer sequences for the target genes are presented in Supplementary Table S2.

### 2.5 Effect of osteogenic capacity in vivo by biomimetic periosteum

All animal experiments were approved by the Ethics Committee of Soochow University. 8-week-old male Sprague-Dawley (SD) rats (250–300 g) were selected, all rats were sourced from Soochow University Laboratory Animal Center, and acclimatized and fed for 1 week before the experiment (Approval No. SUDA20240911A15). They were randomly divided into 4 groups: Defect, GelMA, Exo-GelMA, and BMP-2@Exo-GelMA. After all rats were routinely anesthetized, 2 symmetrical round cranial defects (5 mm in diameter) were created in their skulls. Subsequently, the corresponding biomimetic periosteum was implanted into the defect area according to the grouping, and the Defect group was the control group.

After surgery, rat skull samples were executed and removed at 4 and 8 weeks, and bone volume changes in the bone defect areas were assessed by micro-computed tomography (Micro-CT) scanning to measure the volume of new bone formation and trabecular structure. The analysis included metrics such as bone mineral density (BMD, g/cm^3^) and bone volume ratio (BV/TV, %).

The rat cranial bone samples were fixed, dehydrated, paraffin-embedded and sectioned. The repair of bone tissue and new bone formation were assessed using hematoxylin-eosin (HE) staining and Masson trichrome staining. Immunohistochemical staining was used to assess the role of biomimetic periosteum in modulating inflammation at the site of bone injury and promoting osteogenesis.

### 2.6 Data analysis and statistics

All data were expressed as mean ± standard deviation and statistically analyzed using GraphPad Prism software. Differences between groups were compared using one-way analysis of variance (ANOVA), and differences were considered statistically significant at *P* < 0.05.

## 3 Result

### 3.1 Preparation and characterization of biomimetic periosteum

**FIGURE 2:**
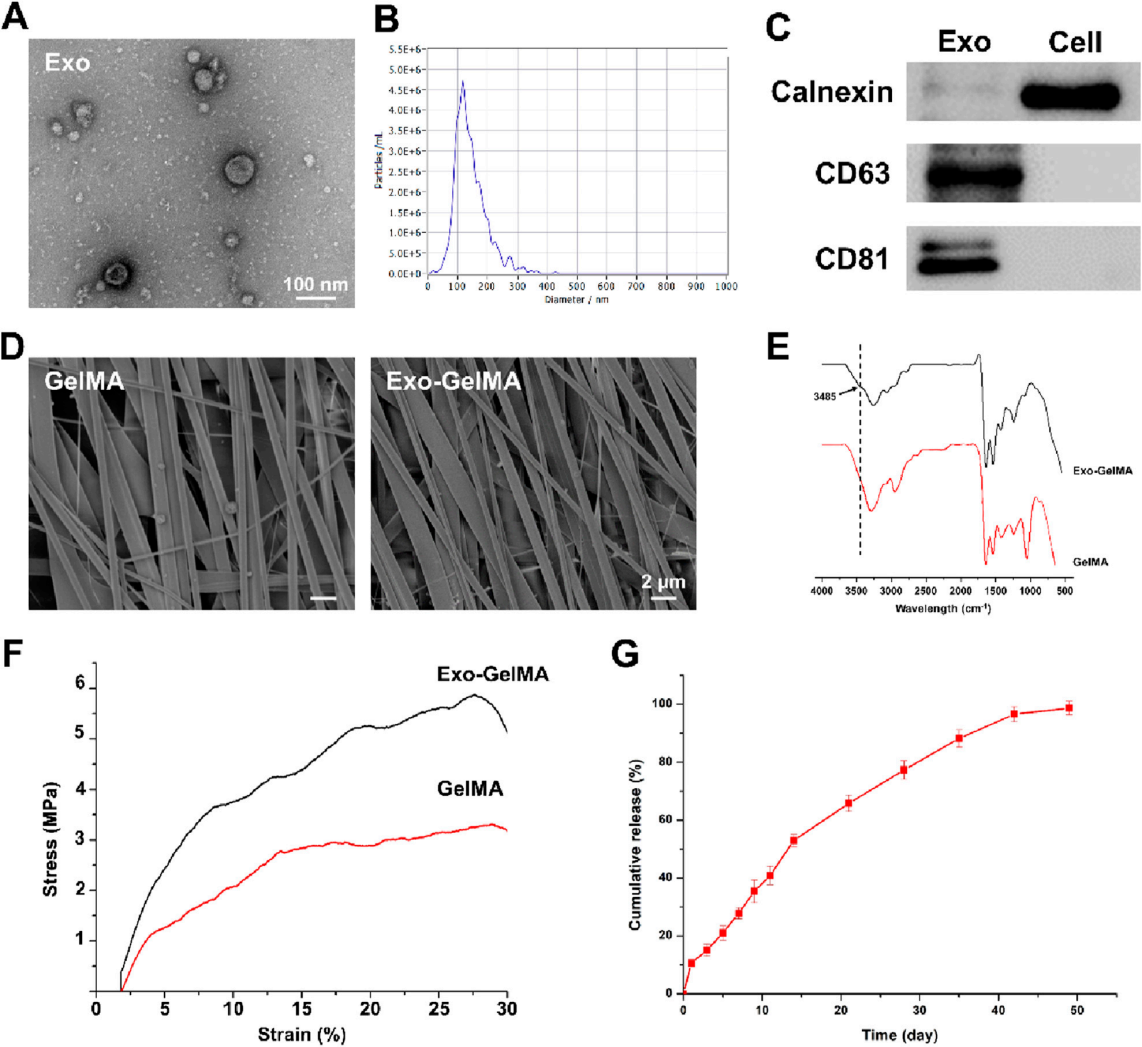
Characterization of exosomes and biomimetic periosteum. **(A)** The representative TEM images of exosomes. **(B)** The size of exosomes. **(C)** Western blot analysis of the exosomes specific protein markers. **(D)** The representative TEM images of GelMA and Exo-GelMA. **(E)** FTIR of GelMA and Exo-GelMA. **(F)** The stress-strain curve of GelMA and Exo-GelMA. **(G)** The release rate of exosomes.

In this study, we designed a biomimetic periosteum by fabricating GelMA membranes through electrospinning and successfully conjugating BMP-2-loaded M2 macrophage exosomes via amidation reaction (Figure 1A). Firstly, to verify the successful extraction and characteristics of exosomes, the results of TEM show that exosomes presented a typical spherical membrane vesicle structure with a diameter of approximately 100 nm, which was in accordance with the expected morphology of exosomes (Figures 2A,B). Additionally, Western blot (WB) was employed to detect the protein expression of exosome markers CD81 and CD63, both showing significant positive signals, further confirming the purity and integrity of exosomes (Figure 2C).

Subsequently, the surface morphology of the biomimetic periosteum was characterized by SEM. The results indicated that the unmodified GelMA membrane exhibited a uniform and dense oriented alignment structure (Figure 2D), and after conjugating exosomes, the fiber alignment remained regular, suggesting that the modification did not significantly affect the structural characteristics of the nanofibers. Moreover, FTIR analysis was conducted to further verify the occurrence of the amidation reaction. The infrared spectrum of Exo-GelMA showed a characteristic amide bond absorption peak at 3,485 cm^−1^, confirming that exosomes were successfully conjugated to the GelMA membrane via chemical bonds (Figure 2E). The results of XPS also indicated that the peak area proportion attributed to carboxyl carbon (O-C=O) in the C1s spectrum of Exo-GelMA significantly decreased compared to GelMA, demonstrating that GelMA and exosomes rich in carboxyl groups (-COOH) and amino groups (-NH_2_) are condensed and bonded through amide bonds (Supplementary Figure S1). Mechanical tests revealed that the tensile strength of Exo-GelMA was slightly higher than that of the GelMA membrane (Figure 2F), which basically met the mechanical requirements for bone tissue engineering applications.

In the drug release experiment, we evaluated the release kinetics of exosomes in the biomimetic periosteum. The results showed that the biomimetic periosteum released approximately 50% of exosomes within the first 2 weeks at a relatively fast rate, and then the release rate gradually slowed down, reaching over 90% cumulative release by day 45 (Figure 2G). This indicated that the biomimetic periosteum had good sustained-release performance, providing a basis for the continuous action of exosomes *in vivo*.

The swelling ratio was measured at equilibrium under physiological conditions (PBS, 37°C), while degradation profiles were assessed over 28 days via mass loss analysis. The results showed that both the GelMA group and the Exo-GelMA group exhibited excellent swelling properties, with no significant differences. However, in terms of degradation performance, the addition of exosomes increased the degradation time of the GelMA membrane (Supplementary Figure S2).

In summary, the above results demonstrated that we successfully prepared a biomimetic periosteum with oriented alignment structure and functional modification characteristics, providing a solid foundation for subsequent *in vitro* and *in vivo* studies.

### 3.2 Biocompatibility of biomimetic periosteum

**FIGURE 3:**
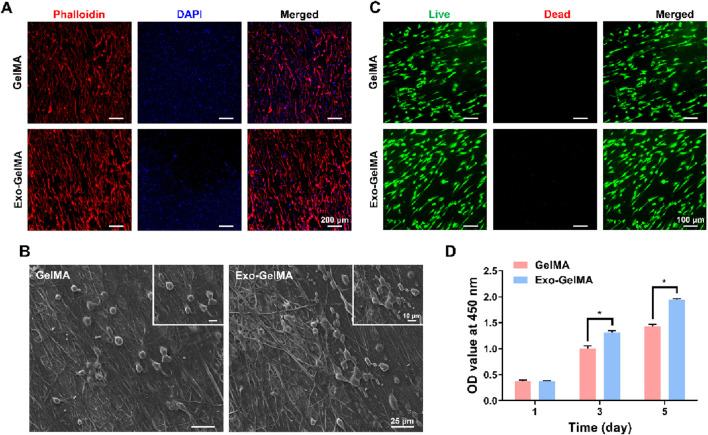
Biocompatibility of biomimetic periosteum. **(A)** Cytoskeletal staining of BMSCs seeded on the surface of biomimetic periosteum after 24 h **(B)** SEM images of cell morphology on the surface of biomimetic periosteum after 24 h. **(C)** Live/dead staining of BMSCs seeded on the surface of biomimetic periosteum after 24 h **(D)** CCK-8 assay indicating the proliferation rate of BMSCs after 1, 3 and 5 days. (*P < 0.05). OD, optical density.

To evaluate the biocompatibility of the biomimetic periosteum, we first observed the morphology of the cells and the adaptability of the periosteum surface. BMSCs were seeded on the surface of the biomimetic periosteum and cytoskeleton immunofluorescence staining and SEM observation were performed. As shown in Figure 3A, the cells in both groups formed uniformly distributed pseudopodia on the surface of the biomimetic periosteum and presented a good stretched and expanded state. The cytoskeleton staining showed a clear fibrous structure. The SEM images further confirmed the good adhesion and interaction between the cells and the surface of the biomimetic periosteum, with a high cell distribution density, indicating that the material has good biocompatibility (Figure 3B).

Next, we observed the growth of cells on the biomimetic periosteum through live/dead cell staining. As shown in Figure 3C, the BMSCs seeded on the biomimetic periosteum began to adhere to the periosteum surface after 24 h of culture. In both groups, almost no dead cell staining signals were observed, indicating that the biomimetic periosteum has good cell compatibility.

The CCK-8 cell proliferation assay further verified the good biocompatibility of the biomimetic periosteum. On the 3rd and 5th days of culture, the cell proliferation rate of the Exo-GelMA group was significantly higher than that of the GelMA group, showing a time-dependent growth trend. Notably, the cell proliferation rate of the Exo-GelMA group on the 5th day was approximately 1.36 times that of the GelMA group (Figure 3D).

### 3.3 The impact of biomimetic periosteum on macrophage polarization

**FIGURE 4:**
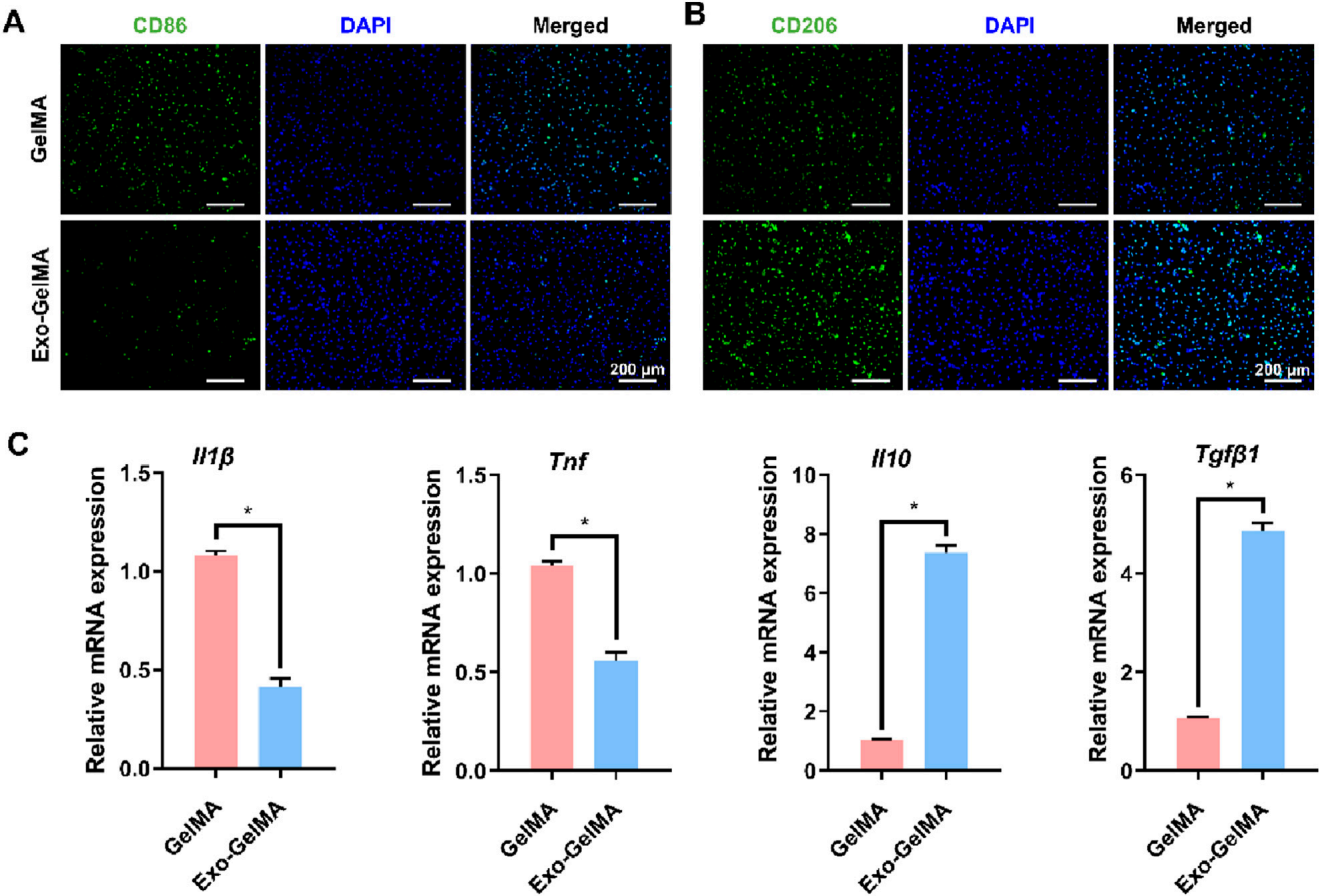
The effect of biomimetic periosteum on the polarization of macrophages *in vitro*. **(A)** Fluorescence microscope images of immunofluorescent staining of CD86 after 24 h. **(B)** Fluorescence microscope images of immunofluorescent staining of CD206 after 24 h **(C)** *Il1β*, *Tnf*, *Il10* and *Tgfβ1* gene expression of macrophages evaluated by qRT-PCR after 24 h *P < 0.05.

To explore the regulatory effect of biomimetic periosteum on the immune microenvironment, we focused on evaluating its influence on macrophage polarization. Immunofluorescence staining results indicated that compared with the GelMA group, the expression of M2 marker CD206 was significantly enhanced in the Exo-GelMA group (Figure 4B), while the expression of M1 marker CD86 was significantly reduced (Figure 4A), suggesting that the biomimetic periosteum loaded with exosomes promoted the transformation of macrophages to the M2 phenotype.

To further verify this effect, we detected the expression levels of M1 and M2 phenotype-related genes by qPCR. The results showed that the expression of *Il-1β* and *Tnf* was significantly downregulated in the Exo-GelMA group, while the expression of *Il10* and *Tgfβ1* was significantly upregulated (Figure 4C). These results indicated that by loading exosomes, the biomimetic periosteum not only inhibited the transformation to the inflammatory M1 phenotype but also effectively promoted the formation of the anti-inflammatory M2 phenotype.

### 3.4 The impact of biomimetic periosteum on osteogenic differentiation

**FIGURE 5:**
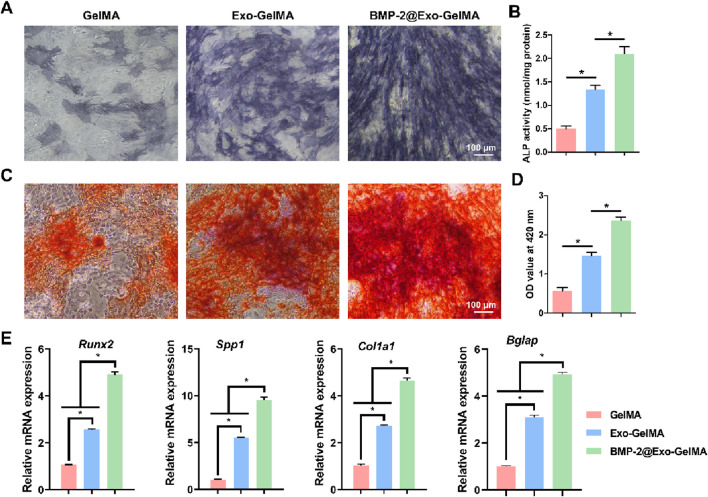
Osteogenic differentiation of BMSCs. **(A)** The represent images of ALP staining after 7 days. **(B)** ALP activity of BMSCs after 7 days. **(C,D)** The represent images and quantitative analysis of ARS after 7 days. **(E)** The expression of osteogenesis-related genes after 7 days *P < 0.05.

75.6% of BMP-2 was loaded into M2 macrophage-derived exosomes. In the osteogenic differentiation experiments, we first evaluated the early osteogenic differentiation ability through alkaline phosphatase (ALP) staining. As shown in Figures 5A,B, the ALP activity in the BMP-2@Exo-GelMA group was the highest, significantly higher than that in the Exo-GelMA group and the GelMA group, indicating that it promoted the early differentiation of BMSCs. Additionally, the ALP activity in the Exo-GelMA group was also significantly higher than that in the GelMA group (Figure 5B).

The alizarin red staining results indicated that the BMP-2@Exo-GelMA group had the highest calcium deposition and the largest positive staining area, which was evenly distributed, suggesting that the addition of BMP-2 further enhanced the osteogenic mineralization ability of the biomimetic periosteum (Figures 5C,D).

Next, we analyzed the expression levels of osteogenesis-related genes, including *Runx2*, *Spp1*, *Col1a1*, and *Bglap*, through PCR experiments. The results showed that the expression of all target genes in the BMP-2@Exo-GelMA group was significantly higher than that in the Exo-GelMA group and the GelMA group (Figure 5E).

These results suggested that the loading of exosomes alone can increase the osteogenic differentiation ability of the biomimetic periosteum to a certain extent, while the biomimetic periosteum loaded with both BMP-2 and exosomes had a stronger osteogenic mineralization ability, providing a solid cellular and molecular basis for bone repair.

### 3.5 *In vivo* osteogenesis ability determination of biomimetic periosteum

**FIGURE 6:**
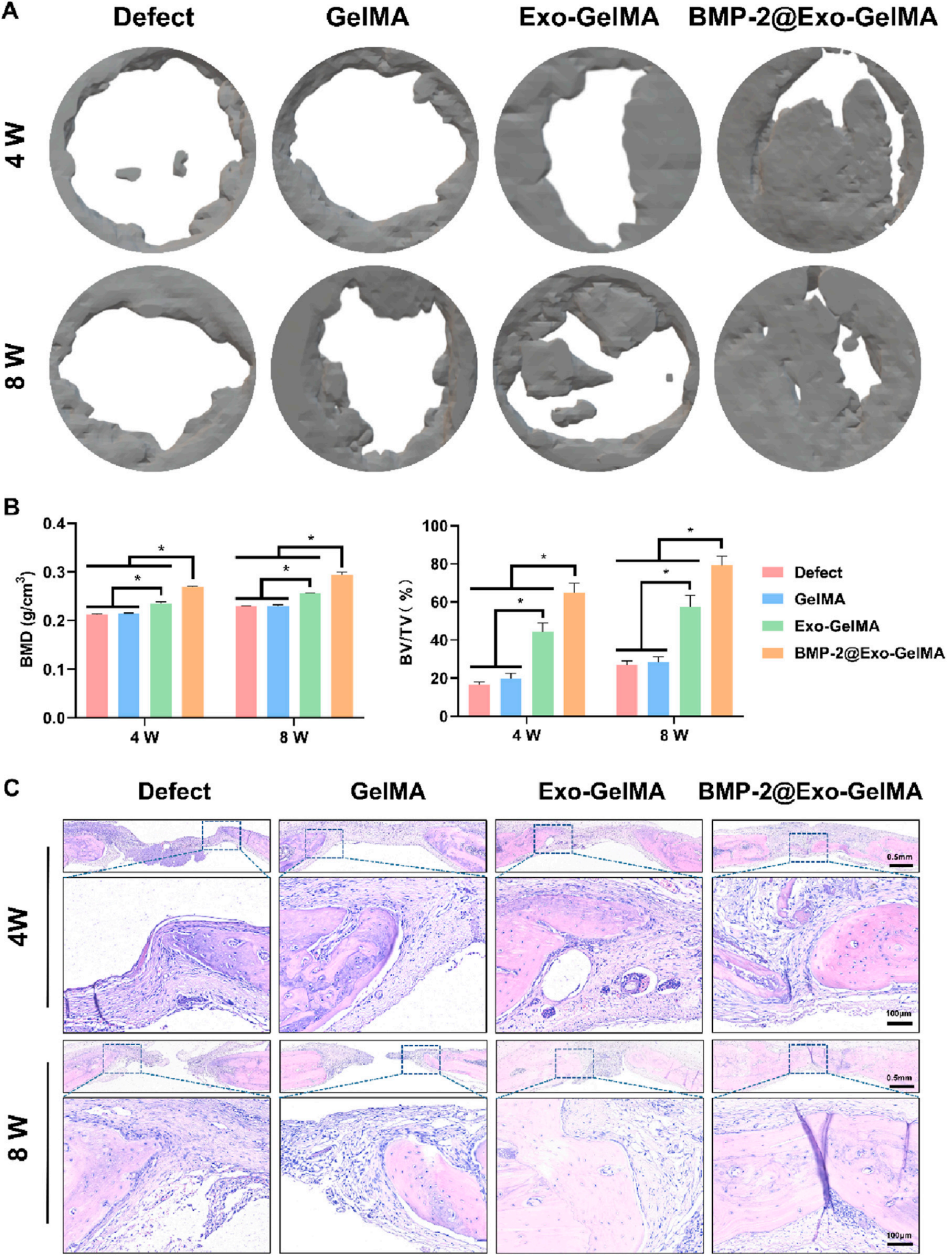
The evaluation of bone defect regeneration *in vivo*. **(A)** The new bone formation in the bone defects evaluated by Micro-CT imaging and 3D reconstruction at 4 and 8 weeks. **(B)** Quantitative analysis of BV/TV and BMD in defect area at 4 and 8 weeks. **(C)** Representative HE staining of the defect area. *P < 0.05.

To further verify the bone injury repair ability of the biomimetic periosteum, we implanted the biomimetic periosteum into the rat cranial defect model and evaluated its osteogenic repair ability through Micro-CT, HE staining, Masson staining and immunohistochemical analysis. As shown in Figure 6A, after 4 and 8 weeks of implantation, only a small amount of new bone tissue was formed in the defect area of the GelMA group. The Exo-GelMA group and the BMP-2@Exo-GelMA group had more newly formed bone tissue than the Defect group and the GelMA group. Especially in the BMP-2@Exo-GelMA group, the cranial defect area was basically repaired completely after 8 weeks of implantation of the biomimetic periosteum. To further assess the newly formed bone tissue, we calculated the BMD and the BV/TV within the defect region, as depicted in Figure 6B. The results demonstrated that the BMD and BV/TV values in the BMP-2@Exo-GelMA group were significantly elevated compared to those in the other experimental groups at both 4 and 8 weeks. These findings suggest that the biomimetic periosteum incorporating BMP-2 and exosomes exhibits a superior capacity to enhance bone defect repair.

The reparative bone tissue was further assessed using HE staining and Masson staining. The outcomes of these staining techniques corroborated the findings from the Micro-CT reconstructions. As illustrated in Figure 6C, minimal bone regeneration was observed in both the Defect and GelMA groups, with the defect areas predominantly occupied by extensive fibrous connective tissue. In contrast, the BMP-2@Exo-GelMA and Exo-GelMA groups exhibited a greater presence of connective tissue and newly formed bone tissue (Figure 6C; Supplementary Figure S3).

**FIGURE 7:**
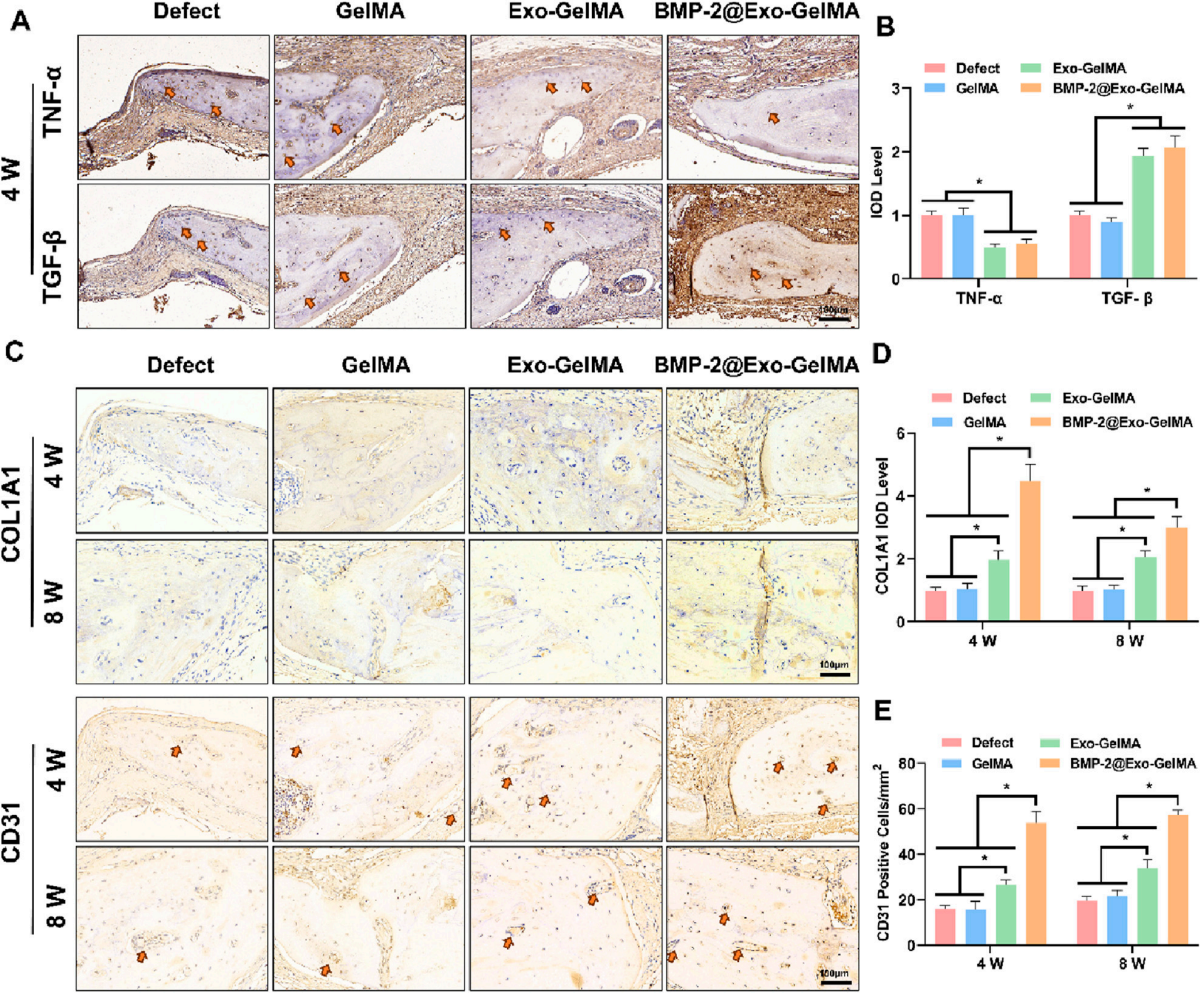
Immunohistochemical analysis. **(A)** Immunohistochemical staining for TNF-α and TGF-β in the defect area at 4 weeks. **(B)** Quantitative analysis of TNF-α and TGF-β‐positive area. **(C)** Immunohistochemical staining for COL1A1 and CD31 in the defect area at 4 and 8 weeks. **(D,E)** Quantitative analysis of COL1A1 and CD31‐positive area at 4 and 8 weeks *P < 0.05.

We evaluated the inflammatory environment in the defect area through TGF-β and TNF-α immunohistochemical staining. As shown in Figures 7A,B, the BMP-2@Exo-GelMA group significantly promoted the expression of TGF-β and inhibited the expression of TNF-α at 4 weeks. This indicates that BMP-2@Exo-GelMA has a better advantage in eliminating local inflammation. In addition, immunohistochemical staining was conducted to assess the expression of osteogenesis-related markers (COL1A1) and angiogenesis-related markers (CD31) in order to evaluate the expression of osteogenic proteins and the angiogenic potential across different experimental groups. As illustrated in Figure 7C, only a limited number of positively stained cells were observed surrounding the defect in both the Defect and GelMA groups. In contrast, sections from the BMP-2@Exo-GelMA and Exo-GelMA groups exhibited a denser and more extensive distribution of COL1A1 and CD31 immunostaining (Figures 7C–E). These findings indicated that the biomimetic periosteum containing BMP-2 and exosomes demonstrated the most pronounced capacity to enhance angiogenesis and bone formation among the four groups studied.

## 4 Discussion

In this study, we successfully constructed a biomimetic periosteum with a directional alignment structure. Specifically, GelMA electrospun membranes were bonded with M2 macrophage exosomes via amidation reaction and loaded with BMP-2 factor. GelMA is widely used in tissue engineering due to its excellent biocompatibility, controllable degradability and good mechanical properties (Lee et al., 2025; Sun et al., 2023b; Li G. et al., 2024). On the one hand, GelMA membranes prepared by electrospinning technology have a directional alignment structure, mimicking the microstructure of natural periosteum. The directional alignment structure of periosteum not only provided sufficient attachment sites for osteoblasts but also promoted the directional growth of cells along the membrane surface through the directional characteristics of the structure, thereby further enhancing the cell migration and differentiation ability during the bone defect repair process (Orwoll, 2003; Lin et al., 2014). On the other hand, by combining growth factors (Yao et al., 2022; Wu et al., 2025), metal ionsor (Li K. et al., 2025) or exosomes (Li X. et al., 2024), GelMA can further improve bone repair ability (Yu et al., 2024; Yang et al., 2023). In this study, we combined M2 macrophage exosomes with GelMA membranes through amidation reaction, which not only retained the biological activity of exosomes but also utilized the miRNA, proteins and other bioactive substances in exosomes to further regulate the local immune microenvironment (Ma et al., 2023). The loading of BMP-2 enhanced the osteoinductive effect of the biomimetic periosteum in bone repair by promoting the differentiation of osteoblasts (Yang et al., 2023). Through this multifunctional design, the biomimetic periosteum could meet the requirements of bone repair in both structure and function, and has broad clinical application prospects.

Macrophages play a crucial role in bone injury repair (Wang et al., 2025). Yang et al. found that porous tantalum can promote the transformation of macrophages to M2 type through the calcium signaling pathway and enhance the proliferation, migration, and osteogenic differentiation of BMSCs through exosomes secreted by M2-type macrophages (Yang et al., 2024). Similarly, the results of this study also showed that biomimetic periosteum can effectively promote the polarization of macrophages to M2 type. This finding is of great significance for the regulation of the immune microenvironment after bone injury. M2-type macrophages are known to have anti-inflammatory and pro-repair effects, secreting various cytokines and growth factors to promote tissue repair and regeneration (Jin S. et al., 2024). In contrast, M1-type macrophages mainly exacerbate inflammation by secreting pro-inflammatory factors and inhibit tissue repair. Our research results indicated that biomimetic periosteum can induce the transformation of macrophages to M2 type through interaction with macrophage exosomes, thereby altering the immune microenvironment after bone injury and promoting the tissue repair process. By modulating the immune microenvironment at the bone injury site, biomimetic periosteum can provide a better foundation for bone tissue regeneration.

The success of bone repair not only depends on the regulation of the immune microenvironment but also requires the differentiation of osteoblasts and the formation of bone matrix (Wang et al., 2025; Li B. et al., 2025). Bionic periosteum plays a role in promoting bone repair through two main mechanisms: on the one hand, bionic periosteum improves the immune microenvironment by inducing the polarization of macrophages to the M2 type, which indirectly promotes the differentiation of BMSCs; on the other hand, in bionic periosteum, the loading of BMP-2 not only enhances osteoinductive function of the periosteum, but also continuously stimulates the differentiation of BMSC through its slow release between cells, effectively promoting the process of bone repair. We evaluated the osteoinductive ability of biomimetic periosteum through alizarin red staining, alkaline phosphatase staining, and PCR detection. The results showed that biomimetic periosteum could not only significantly promote the expression of osteogenesis-related genes such as *Runx2* and *Spp1* but also accelerate the transformation of osteoblasts to mature osteoblasts. Therefore, biomimetic periosteum significantly enhances its effect in bone injury repair through dual mechanisms of immune regulation and factor release. Finally, we implanted biomimetic periosteum into rat cranial bone defect models. The results of Micro-CT and histological staining showed that biomimetic periosteum significantly promoted the repair of bone defects. These experimental results not only verified the role of biomimetic periosteum in promoting bone tissue regeneration but also provided strong support for its potential in clinical applications.

## 5 Conclusion

The biomimetic periosteum constructed in this study demonstrated excellent performance in promoting bone defect repair. By combining the biocompatibility of GelMA, the immunomodulatory effect of exosomes, and the osteoinductive effect of BMP-2, it achieved multi-level support for bone repair. This biomimetic periosteum can not only improve the immune microenvironment at the bone injury site and promote the differentiation of osteoblasts but also accelerate the deposition and mineralization of bone matrix, ultimately achieving effective repair of bone defects. In the future, with the continuous development of materials science and biomedical technology, biomimetic periosteum is expected to become an important therapeutic approach in the field of bone repair.

## Data Availability

The original contributions presented in the study are included in the article/Supplementary Material, further inquiries can be directed to the corresponding authors.
