# Supplementary material for: Biomimetic periosteum combining BMP-2-loaded M2 macrophage-derived exosomes for enhanced bone defect repair
Source: Front Bioeng Biotechnol. 2025 Aug 1;13:1639394. doi: 10.3389/fbioe.2025.1639394 (PMC12354627; doi:10.3389/fbioe.2025.1639394)
Supplement: Supplementary file 1 [file Supplementaryfile1.docx]

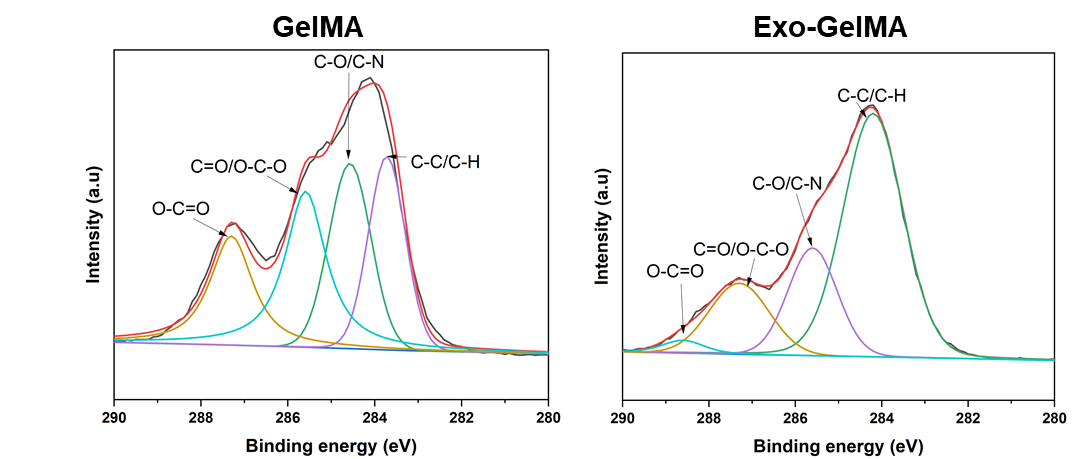


**Supplementary Fig. 1.** XPS measurement of GelMA and Exo-GelMA.


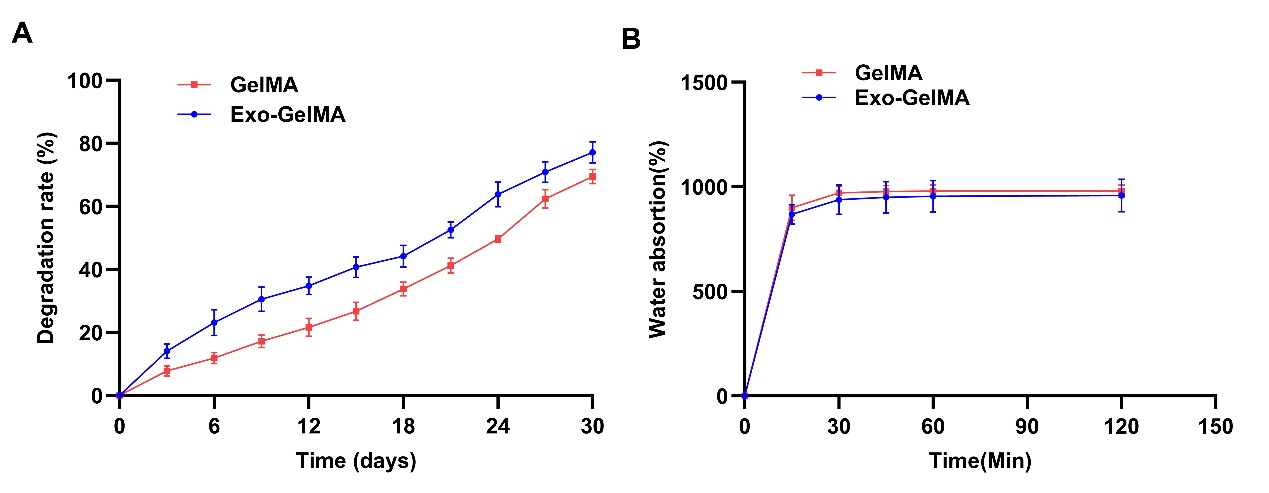


**Supplementary Fig. 2.** (A) Degradation rate of GelMA and Exo-GelMA. (B) Water absortion of GelMA and Exo-GelMA.


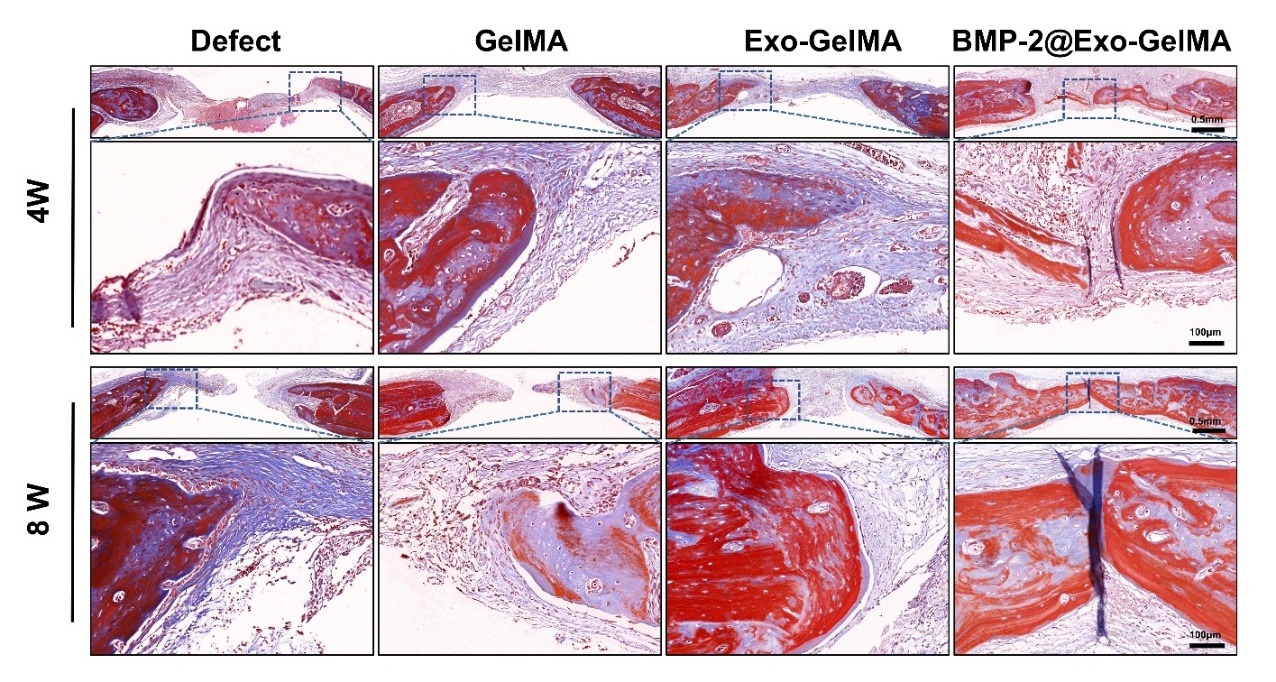


**Supplementary Fig. 3.** Masson’s trichrome staining of new bone at 4 and 8 weeks.

**Supplementary Table 1.** Primer sequences for mouse BMMs.

| Gene | Forward primer sequence (5ʹ -3ʹ) | Reverse primer sequence (5ʹ -3ʹ) |
| --- | --- | --- |
| *Gapdh* | GGTTGTCTCCTGCGACTTCA | GGTCCAGGGTTTCTTACTCC |
| *Tgfβ1* | CCAGATCCTGTCCAAACTAAGG | CTCTTTAGCATAGTAGTCCGCT |
| *Il10* | TTCTTTCAAACAAAGGACCAGC | GCAACCCAAGTAACCCTTAAAG |
| *Tnf* | ATGTCTCAGCCTCTTCTCATTC | GCTTGTCACTCGAATTTTGAGA |
| *Il1β* | CACTACAGGCTCCGAGATGAACAAC | TGTCGTTGCTTGGTTCTCCTTGTAC |

**Supplementary Table 2.** Primer sequences for rat BMSCs.

| Gene | Forward primer sequence (5ʹ -3ʹ) | | Reverse primer sequence (5ʹ -3ʹ) |
| --- | --- | --- | --- |
| *Gapdh* | GACATGCCGCCTGGAGAAAC | | AGCCCAGGATGCCCTTTAGT |
| *Bglap* | AACGGTGGTGCCATAGATGC | | AGGACCCTCTCTCTGCTCAC |
| *Col1a1* | CAGGCTGGTGTGATGGGATT | CCAAGGTCTCCAGGAACACC | |
| *Spp1* | GCGGTTCACTTTGAGGACAC | TATGAGGCGGGGATAGTCTTT | |
| *Runx2* | ATCCAGCCACCTTCACTTACACC | GGGACCATTGGGAACTGATAGG | |
